# Expression and regulation of α-transducin in the pig gastrointestinal tract

**DOI:** 10.1111/jcmm.12026

**Published:** 2013-02-18

**Authors:** Maurizio Mazzoni, Roberto Giorgio, Rocco Latorre, Claudia Vallorani, Paolo Bosi, Paolo Trevisi, Giovanni Barbara, Vincenzo Stanghellini, Roberto Corinaldesi, Monica Forni, Maria S Faussone-Pellegrini, Catia Sternini, Paolo Clavenzani

**Affiliations:** a Department of Veterinary Medical Science, University of BolognaItaly; b Department of Medical and Surgical Sciences, University of BolognaItaly; c Department of Agri-food Protection and Improvement, University of BolognaItaly; d Department of Anatomy, Histology and Forensic Medicine, University of FlorenceFirenze, Italy; e CURE/DDRC, Division of Digestive Diseases, Departments Medicine and Neurobiology, David Geffen School of Medicine, UCLA, Los Angeles, and Veterans Administration Greater Los Angeles Health SystemCA, USA; f Centro Interdipartimentale di Ricerche sull'Alimentazione Umana (CIRAU), University of BolognaItaly

**Keywords:** α-gustducin, taste receptors, enteroendocrine cells, chemosensing

## Abstract

Taste signalling molecules are found in the gastrointestinal (GI) tract suggesting that they participate to chemosensing. We tested whether fasting and refeeding affect the expression of the taste signalling molecule, α-transducin (G_αtran_), throughout the pig GI tract and the peptide content of G_αtran_ cells. The highest density of G_αtran_-immunoreactive (IR) cells was in the pylorus, followed by the cardiac mucosa, duodenum, rectum, descending colon, jejunum, caecum, ascending colon and ileum. Most G_αtran_-IR cells contained chromogranin A. In the stomach, many G_αtran_-IR cells contained ghrelin, whereas in the upper small intestine many were gastrin/cholecystokinin-IR and a few somatostatin-IR. G_αtran_-IR and G_αgust_-IR colocalized in some cells. Fasting (24 h) resulted in a significant decrease in G_αtran_-IR cells in the cardiac mucosa (29.3 ± 0.8 *versus* 64.8 ± 1.3, *P* < 0.05), pylorus (98.8 ± 1.7 *versus* 190.8 ± 1.9, *P* < 0.0 l), caecum (8 ± 0.01 *versus* 15.5 ± 0.5, *P* < 0.01), descending colon (17.8 ± 0.3 *versus* 23 ± 0.6, *P* < 0.05) and rectum (15.3 ± 0.3 *versus* 27.5 ± 0.7, *P* < 0.05). Refeeding restored the control level of G_αtran_-IR cells in the cardiac mucosa. In contrast, in the duodenum and jejunum, G_αtran_-IR cells were significantly reduced after refeeding, whereas G_αtran_-IR cells density in the ileum was not changed by fasting/refeeding. These findings provide further support to the concept that taste receptors contribute to luminal chemosensing in the GI tract and suggest they are involved in modulation of food intake and GI function induced by feeding and fasting.

## Introduction

Sensing of luminal contents by the gastrointestinal (GI) tract mucosa plays a critical role in the control of digestion, absorption, food intake and metabolism 1, 2 by triggering functional responses appropriate for beneficial or potentially harmful substances. Enteroendocrine (EEC) cells act as specialized transducers of luminal content, by releasing signalling molecules, which activate nerve fibres as well as local and distant targets to influence gut functions. EECs can be either ‘open-type’ or ‘closed-type’ depending on their microvilli reaching or not the lumen 1–3. Both types of cells can be regulated by intraluminal content, either directly (‘open cells’) or indirectly (‘closed cells’) through neural and humoural mechanisms to release a variety of secretory products, including gastrin (G cells), ghrelin (P or X cells), somatostatin (D cells), cholecystokinin (CCK) (I cells), serotonin (enterochromaffin cells), glucose-dependent insulinotropic peptide (GIP) (K cells), glucagon-like peptides (GLPs) and peptide YY (PYY) (L cells), according to the different substances detected in the lumen 1–3. Once released, these signalling molecules affect different functions ranging from gastrointestinal motility and secretion to feeding regulation *via* the brain-gut axis 1–3.

The discovery that taste receptors (TRs) and signalling molecules identified in the oral cavity are expressed in the GI mucosa, suggests that they play a role in chemosensing in the gut. TRs are G protein-coupled receptors (GPCRs) sensing bitter (T2Rs), or sweet and umami (T1Rs) tastes. T2Rs are a large family of receptors (25–36 in mammals) perceiving a multitude of tastants, whereas T1Rs comprise 3 receptors that heterodimerize to sense sweetness (T1R2 and T1R3) or umami (T1R1 and T1R3) 4–6. T1Rs and T2Rs mediate gustatory signalling by interacting with specific Gα subunits, including α-gustducin (G_αgust_) and α-transducin (G_αtran_) 7 through the activation of different effector systems leading to intracellular Ca^2+^ increase and transmitter release. G_αgust_ or G_αtran_ immunoreactivity (IR) has been localized to epithelial EECs and non-EECs in the rodent 8–11, pig 12, 13 and human 14 GI tract and pancreatic duct 15.

The aims of this study were to characterize the cellular sites of expression of G_αtran_ and test the hypothesis that G_αtran_ is modulated by fasting and refeeding in the GI tract of the pig, an animal model closer to humans compared with rodents for food intake, body size, lifespan and body proportion.

## Materials and methods

Large White male pigs (*n* = 12), of about 45 days of age with an average weight of 12.0 ± 0.3 kg, purchased from Suidea (Reggio Emilia, Italy), were fed with a standard balanced diet and housed individually in pens with a mesh floor in a temperature-controlled room and tap water freely available. Following 1 day adaptation, animals were divided into three groups: standard diet (control, *n* = 4), fasted for 24 h (fasted, *n* = 4) and refed for 24 h after fasting (refed, *n* = 4). Experimental procedures were approved by the Ethic Committee for Experimental Animals of the University of Bologna, Italy.

Pigs were deeply anaesthetized with sodium thiopental (10 mg/kg body weight, Zoletil 100, Virbac) and killed by an intracardiac injection of Tanax^**®**^ (0.5 ml/kg BW; Intervet Italia). Specimens of the GI tract: oesophagus (cervical, thoracic and abdominal tract), stomach (cardiac, near to the gastric diverticulum; oxyntic, in the greater curvature; and pyloric, close to the pyloric sphincter), duodenum (about 10 cm from the pyloric sphincter), middle jejunum and ileum, caecum, ascending colon (near the centrifugal turns), descending colon (about 25 cm from the anus) and rectum (in the *ampulla recti*) were collected, pinned flat on balsa wood, fixed in 10% buffered formalin for 24 h at room temperature (RT), dehydrated and embedded in paraffin.

### Immunohistochemistry

Serial (5 μm thick) sections mounted on poly-L-lysine–coated slides were processed for single and double labelling immunofluorescence using antibodies directed to G_αtran_ or G_αgust_, chromogranin A (CgA), a generalized marker for EECs, or specific markers for EEC subtypes (ghrelin, GHR, somatostatin, SOM and gastrin/cholecystokinin GAS/CCK) (Table 1). Briefly, sections were deparaffinized through graded ethanols to xylene, rehydrated and heated in sodium citrate buffer (pH 6.0) in a microwave (2 cycles at 800 W, 5 min each) for antigen unmasking. Sections were incubated in 15% normal horse serum/0.01 M phosphate buffer saline (PBS) (1 h at RT) to prevent non-specific staining, followed by primary antibodies in PBS (overnight) and a mixture of fluorescein isothiocyanate (FITC)-conjugated, tetramethyl rhodamine isothiocyanate (TRITC)-conjugated, Alexa Fluor® 594- and Alexa Fluor® 488-conjugated secondary antibodies all diluted in PBS (Table 1), then coverslipped with buffered glycerol, pH 8.6. As the antibodies to G_αtran_ and G_αgust_ were generated in the same species, serial sections (3 μm thick) were used to test their colocalization.

**Table 1:** List and dilution of primary and secondary antibodies

Primary antibodies	Code	Species	Dilution	Supplier
α-Transducin	sc-390	rabbit	1:600	Santa Cruz
α-Gustducin	sc-395	rabbit	1:500	Santa Cruz
Chromogranin A	MON9014	mouse	1:1000	Monosan
Gastrin/Cholecystokinin	GAS/CCK#9303	mouse	1:1000	CURE/DDC
Ghrelin	sc-10368	goat	1:800	Santa Cruz
Somatostatin	S6	mouse	1:1000	CURE/DDC

Secondary antisera	Dilution	Supplier
Alexa 594-conjugated goat anti-mouse IgG	1:800	Mol. Probes
FITC-conjugated goat anti-rabbit IgG	1:500	Calbiochem
TRITC-conjugated donkey anti-rabbit IgG	1:500	Jackson
Alexa 488-conjugated donkey anti-goat IgG	1:800	Mol. Probes

CURE/DDC, UCLA, Los Angeles, CA, USA; Chemicon International, Temecula, CA, USA; Monosan, Sanbio B.V. Frontstraat, Uden, the Netherlands; Santa Cruz Biotecnology, Inc., CA, USA.); Calbiochem-Novabiochem Corporation, San Diego, CA, USA; Molecular Probes, Eugene, OR., USA; Jackson ImmunoResearch Laboratories, Inc., West Grove, PA, USA).

### Specificity of antibodies

Specificity of G_αtran_, G_αgust_ and GAS/CCK antibodies has been tested by Western blot (Supplementary material) whereas specificity of CgA monoclonal antibody (clone LK2H10) has been previously reported 16. GHR antibody specificity was assessed by pre-adsorption with an excess of the homologous peptide (sc-10368 P, Santa Cruz, CA, USA) or another ghrelin peptide (code 031-52; Phoenix Pharmaceuticals, Inc., Burlingame, CA, USA). The pattern obtained with our S6 SOM antibody completely overlapped with that of SOM rabbit polyclonal antiserum (Monosan SANBIO B.V., Uden, The Netherlands-catalogue PS 204), whose specificity was shown by immunoblocking in the pig gut and pancreas.

### Cell counting and statistical analysis

Cell counting was performed with a 40 × objective lens using a Zeiss Axioplan microscope (Carl Zeiss, Oberkochen, Germany) with appropriate filter cubes to discriminate different wave fluorescence, images were collected with a Polaroid DMC digital photocamera (Polaroid, Cambridge, Mass., USA) and minimal adjustment to brightness and contrast was performed with Corel Photo Paint and Corel Draw (Corel, Dublin, Ireland). Cell counting was performed in a blind fashion by two investigators.

For each piglet, G_αtran_-IR cells were counted in 36 random microscope fields (each field, 0.28 mm^2^), for a total area of 10 mm^2^, in the cardiac, oxyntic and pyloric mucosa, in 50 random villi and glands in the small intestine, and in 50 crypts in the colon. Only villi/glands/crypts located perpendicularly to the mucosal surface were counted. The values were pooled for each experimental group (control, fasted and refed respectively) and, subsequently, the mean and the percentage were calculated. Values were expressed as mean ± standard deviation (SD). Data were analysed using anova One-Way (Graph Prism 4, GraphPad Software, Inc., La Jolla, CA, USA). Statistical significance was determined using the Student's *t*-test. A *P* < 0.05 was considered statistically significant.

## Results

### Distribution of G_αtran_-IR cells in the GI tract

**Fig. 1:**
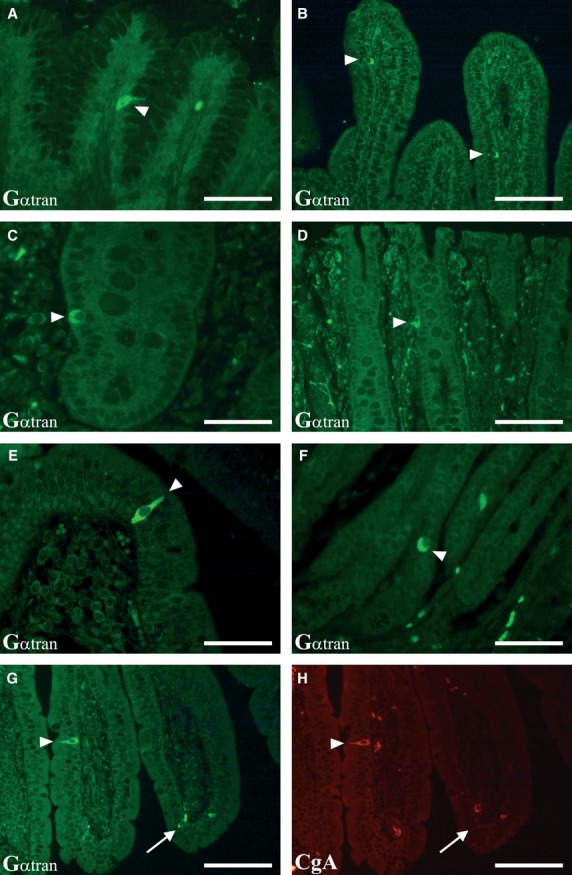
Localization of G_αtran_-IR in the pig GI tract. **A–D** show images of G_αtran_-IR cells in the pyloric (**A**), jejunum (**B**), caecum (**C**) and rectum (**D**) mucosa (arrowheads). G_αtran_-IR cells have the morphology of open-type enteroendocrine cells in the top of the villi of the duodenum (**E**, arrowhead) and of closed-type enteroendocrine cells in the pyloric mucosa (**F**, arrowheads). The bottom images show a G_αtran_-IR enteroendocrine cell expressing chromogranin A (CgA) (**G** and **H**, respectively; arrowheads); the arrow in G and H indicates a G_αtran_-IR cell (**G**) not containing CgA-IR (**H**). A, C, E and F: scale bars = 50 μm; B, D, G and H: scale bars = 100 μm.

G_αtran_-IR cells were detected throughout the whole pig GI tract (Fig. 1A–G), except the oesophagus and oxyntic mucosa. In the pylorus, intense G_αtran_-IR was observed in the basal portion of the gastric gland and in the epithelial lining of the mucosal folds (Fig. 1A and F); G_αtran_-IR cells had elongated, ‘bottle-like’, morphology with homogenously labelled cytoplasm (Fig. 1E and G). In the small intestine, a subset of cells along the crypt-villus axis showed G_αtran_-IR (Fig. 1B, E and G), whereas in the large intestine, labelled cells were generally located in the surface and glandular epithelium (Fig. 1C and D). Most G_αtran_-IR cells had two thin cytoplasmic prolongations, one extending to the endoluminal mucosal surface (Fig. 1E and G) and one to the basal lamina, suggesting they are ‘EEC open-type’ cells 1, 3. In the cardiac and pyloric mucosa, some cells were confined to the basal lamina and did not reach the lumen (Fig. 1F), like ‘EEC closed-type’ cells 1, 3.

### Distribution of the G_αtran_-IR cells in different experimental groups

**Fig. 2:**
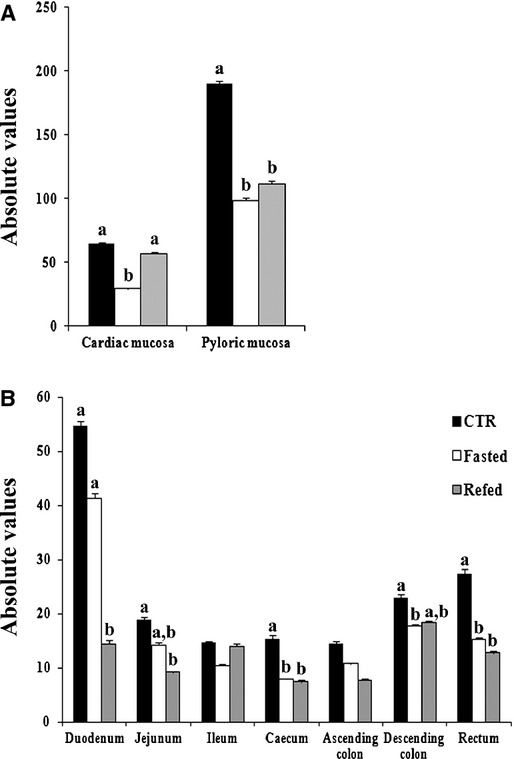
Graphs in **A** and **B** indicate the mean number of G_αtran_-IR cells in the different segments of the pig GI tract. Controls, fasted and refed are denoted as black, white and grey bars respectively. Different letters indicate a significant (*P* < 0.05) statistical difference among groups. Values are expressed as mean ± SD.

In the stomach, the highest density of G_αtran_-IR cells was in the pylorus (there was an average of about 18.9 cells/mm^2^ or 5.3 cells per field); in the small intestine, the highest density of G_αtran_-IR cells was in the duodenum followed by the jejunum and ileum, whereas in the large intestine it was in the rectum followed by descending colon, caecum and ascending colon (Fig. 2A and B). There was a decrease in the density of G_αtran_-IR cells in fasted animals, which was significant in the cardiac mucosa (29.3 ± 0.8 *versus* 64.8 ± 1.3, *P* < 0.05 *versus* control), pylorus (98.8 ± 1.7 *versus* 190.8 ± 1.9, *P* < 0.0 l), caecum (8 ± 0.01 *versus* 15.5 ± 0.5, *P* < 0.01), descending colon (17.8 ± 0.3 *versus* 23 ± 0.6, *P* < 0.05) and rectum (15.3 ± 0.3 *versus* 27.5 ± 0.7, *P* < 0.05), but not in the other regions. Interestingly, refeeding restored the control level of G_αtran_-IR cells in the cardiac mucosa (57 ± 1 *versus* 29.3 ± 0.8 in fasted, *P* < 0.01), but not in the pylorus, caecum, descending colon and rectum where the number of G_αtran_-IR cells in refed was comparable to fasted pigs. In the jejunum, G_αtran_-IR cells in the refed group were less than in the fasted condition and were significantly lower than in controls (9.3 ± 0.2 in refed *versus* 19 ± 0.3 in control, *P* < 0.01). In the ileum and ascending colon, the number of G_αtran_-IR cells in fasted and refed animals was comparable to controls.

### G_αtran_/CgA in the GI tract

The majority of G_αtran_-IR cells co-expressed CgA: 99% of the G_αtran_-IR cells in the cardiac and pyloric mucosa were immunopositive for CgA, whereas 83% and 98% of G_αtran_-IR cells were immunopositive for CgA in the small and large intestine respectively. However, some cells were G_αtran_-IR, but CgA negative (Fig. 1 G and H). In the stomach, G_αtran_-IR/CgA-IR cells were numerous in the glandular epithelium.

The mean numbers of G_αtran_/CgA-IR cells throughout the pig gut are reported in Table 2A. In the cardiac mucosa, the mean number of G_αtran_/CgA-IR cells in control and refed groups is higher than that of fasted group (*P* < 0.05). In the pyloric mucosa, the mean number of G_αtran_/CgA-IR cells in fasted and refed groups was lower than control (control *versus* fasted and control *versus* refed, *P* < 0.05). A general decrease in G_αtran_/CgA-IR cells was observed in the small and large intestine in fasted and refed compared with control. Specifically, in the duodenum and jejunum, the G_αtran_/CgA-IR cells were significantly decreased in refed compared with control (*P* < 0.05). Moreover, in the duodenum, we found a reduced number of G_αtran_/CgA-IR cells in refed compared with fasted (*P* < 0.05). G_αtran_/CgA-IR cells were more abundant in the caecum, descending colon and rectum of control group compared with fasted (*P* < 0.05), whereas in the caecum and in the rectum, refed showed a number of G_αtran_/CgA-IR lower than control (*P* < 0.05). The percentage of the G_αtran_ on the total of CgA-IR cells have been indicated in Table 2B. Furthermore, there were no statistically significant differences in the absolute numbers of CgA-IR cells in the gastric and intestinal mucosa among the three experimental groups.

**Table 2:** (A) Mean number of G_αtran_/CgA-IR cells in the pig GI tract. (B) Percentage of G_αtran_/total CgA-IR cells in the pig GI tract

	*Cardiac mucosa	*Pyloric mucosa	Duodenum	Jejunum	Ileum	Caecum	Ascending colon	Descending colon	Rectum
(A)									
Control	64.5 ± 1.2^a^	190.8 ± 1.9^a^	46.8 ± 0.8^a^	15.8 ± 0.3^a, b^	11.8 ± 0.3	15.3 ± 0.5^a^	13.8 ± 0.5	23 ± 0.6^a^	27.5 ± 0.7^a^
Fasted	29 ± 0.8^b^	96.8 ± 1.7^b^	36 ± 0.9^a^	12 ± 0.4^b, c^	7 ± 0.2	8 ± 0.1^b^	10.5 ± 0.2	17.5 ± 0.3^b^	15.3 ± 0.3^b^
Refed	56 ± 1^a^	111.8 ± 1.9^b^	11 ± 0.6^b^	8 ± 0.2^c^	13.3 ± 0.6	6.8 ± 0.3^b^	7.8 ± 0.2	18.5 ± 0.3^a, b^	12.8 ± 0.3^b^
(B)									
Control	19% (258/1351)	33.7% (763/2262)	49% (187/381)	36.2% (63/174)	27% (47/174)	50.4% (61/121)	40.4% (55/136)	16.5% (92/557)	20.3% (110/543)
Fasted	7% (116/1642)	16% (387/2399)	41.4% (144/348)	23.9% (48/201)	21.9% (28/128)	24.4% (32/131)	30.7% (42/137)	21% (70/333)	17% (61/359)
Refed	15.7% (224/1429)	17.6% (447/2546)	23% (44/191)	18.4% (32/174)	31.2% (53/170)	32.5% (27/83)	27.7% (31/112)	17% (74/434)	12% (51/426)

* Values refer to a total area of 10 mm^2^ for each group. The other values represent the percentage evaluated in 50 villi and in 50 intestinal glands for each group, respectively.

Values with different superscripts within the same column differ significantly (*P* < 0.05).

### G_αtran_/GHR in the gastric mucosa

**Fig. 3:**
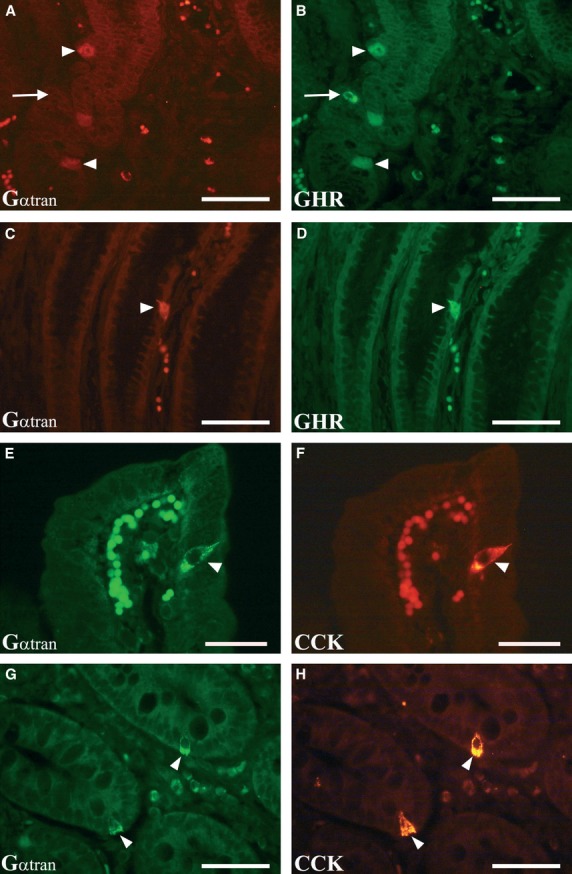
Colocalization of G_αtran_-IR (**A**, **C**, **E** and **G**, arrowheads) with ghrelin (GHR) in the pyloric mucosa (**B** and **D**, arrowheads) and cholecystokinin (CCK) in the jejunum (**F** and **H**, arrowheads). In general, the G_αtran_/GHR-labelled cells were found lying close to the basal lamina of the glands (typical closed-type morphology) (**A** and **B**, arrowheads); the arrows in **A** and **B** indicate a GHR-IR cell (**B**) not containing G_αtran_-IR (**A**). In some cases, G_αtran_/GHR-IR cells were observed in the surface epithelium (typical open-type morphology) (**C** and **D**, arrowheads). The G_αtran_/CCK immunopositive cells were observed in the villi (**E** and **F**, arrowheads) and in the intestinal gland of the jejunum (**G** and **H**, arrowheads). A, B, C, D, G and H: scale bars = 50 μm; E and F: scale bars = 30 μm.

G_αtran_/GHR-IR cells were numerous in the pylorus, from the neck to the base of the glands (Fig. 3A and B), and less abundant in cardiac glands (Fig. 3C and D). Most G_αtran_/GHR cells were ‘closed-type’, lying at the gland basal lamina. Few G_αtran_/GHR-IR cells in the surface epithelium were ‘open-type’ (Fig. 3C and D). In the cardiac and pyloric mucosa, approximately 96% and 91% of G_αtran_-IR cells, respectively, co-expressed GHR. G_αtran_/GHR-IR cells were significantly reduced in fasted *versus* control pigs in both cardiac mucosa (*P* < 0.01) and pylorus (*P* < 0.05). In refed, they were partly restored in the cardiac mucosa (*P* < 0.05), but not pylorus. The mean number and percentage of the G_αtran_ on the total of GHR-IR cells are reported in Table 3. In the cardiac mucosa, the number of GHR-IR cells decreased in fasted *versus* control (114.8 ± 29.4 *versus* 244.5 ± 71.3, *P* < 0.01), while it increased in refed *versus* fasted (241.3 ± 57.5 *versus* 114.8 ± 29.4, *P* < 0.01). There were no statistically significant differences in the mean numbers of GHR-IR cells in the pyloric mucosa among the three experimental groups.

**Table 3:** Mean number and percentage of the colocalized G_αtrans_/total GHR-IR cells in the cardiac and pyloric mucosa

	Cardiac mucosa		Pyloric mucosa	
Control	84.8 ± 1.6^a^	46% (339/735)	168.8 ± 2.6^a^	60.6% (675/1113)
Fasted	26.8 ± 0.8^b^	23.3% (107/459)	97.5 ± 1.7^b^	46.9% (390/831)
Refed	49.8 ± 1^a^	23.2.7% (199/857	107.5 ± 1.8^a, b^	41.5% (430/1036)

Values with different superscripts within the same column indicate statistical significance (*P* < 0.05).

### Colocalization of G_αtran_ with CCK, SOM and G_αgust_ in the duodenum and jejunum

**Fig. 4:**
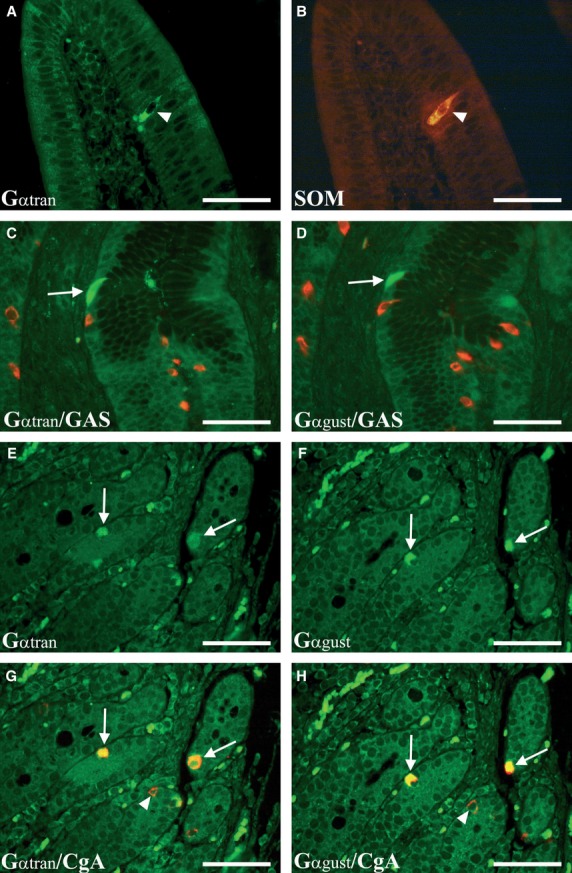
Enteroendocrine cells of the duodenum co-expressing G_αtran_ and SOM-IR (**A** and **B**, arrowheads). Some cells co-expressing G_αtran_/G_αgust_-IRs (**C** and **D**, arrows) (in green) were observed in the pyloric mucosa; these cells were negative for gastrin (GAS-IR) (in red). Photomicrographs **E** and **F** show co-expressing G_αtran_- and G_αgust_-IR enteroendocrine cells (in green) (arrows) in serial sections of the duodenum. The G_αtran_ and G_αgust_ colocalization is readily visible in **G** and **H** (merged images) with chromogranin A (CgA) (arrows) labelled by the red fluorochrome (arrowheads). A–H: scale bars = 50 μm.

Co-expression of G_αtran_ and CCK was observed in open-type cells in the surface and glandular epithelium of the jejunum (Fig. 3E–H). As our monoclonal antibody cannot discriminate CCK and GAS, we could not assess the actual number of GAS and CCK-IR cells in the duodenum where both cell types are present. Few G_αtran_/SOM cells (about 1 positive cell/400 villi) were detected (Fig. 4A and B). The mean number and percentage of the G_αtran_ compared with the total number of CCK-IR cells are reported in Table 4. In the jejunum, approximately 59% of G_αtran_ -IR cells co-expressed CCK. G_αtran_/CCK-IR cells were reduced in fasted and refed compared with controls (*P* < 0.01) in the jejunum. G_αtran_/CCK-IR cells were not visualized in the pylorus and cardiac mucosa (Fig. 4C and D). Finally, occasional G_αtran_/G_αgust_-IR cells were detected in the pylorus (Fig. 4C and D) and duodenum (Fig. 4E–H), which expressed CgA-IR (Fig. 4G and H). Furthermore, the number of CCK-IR cells decreased in fasted *versus* control (19.3 ± 2.5 *versus* 10.3 ± 1, P < 0.01), while no changes were observed in refed *versus* fasted and control groups.

**Table 4:** Mean number and percentage of the colocalized G_αtran_/total CCK-IR cells in the jejunum

Control	13.5 ± 0.3^a^	70% (54/77)
Fasted	8.8 ± 0.2^b^	85.4% (35/41)
Refed	9.5 ± 0.2^b^	71.7% (38/53)

Values with different superscripts within the same column indicate statistical significance (*P* < 0.05).

## Discussion

Taste receptors are likely to represent an important mechanism for sensing nutrients and non-nutrients in the GI lumen and contribute to the initiation of appropriate physiological response of digestion/absorption of nutrients or elimination of harmful substances *via* activation of neuronal and endocrine pathways. We showed that (a) G_αtran_ cells are distributed throughout the GI tract in the pig, a commonly used animal model for studies of human GI physiology and ingestive behaviour, with the exception of the oesophagus and the oxyntic mucosa, (b) most G_αtran_ cells are EEC of the ‘open’ type, (c) many G_αtran_ cells contain GHR in the stomach and CCK in the small intestine, whereas a few contain SOM in the upper bowel, (d) some G_αtran_ cells contained G_αgust_, and (e) fasting and refeeding changed the density of G_αtran_-IR cells, effect that was statistically significant *versus* controls in most, but not all gut regions. These findings support the concept that TRs participate to chemosensing processes controlling multiple GI functions, including food intake and metabolism.

Our results expand previous reports of G_αtran_ or G_αgust_ in the rodent 3, 8–11, 17, pig 12, 13 and human 14 GI mucosa by showing a systematic analysis and characterization of mucosal cells expressing G_αtran_ in the pig intestine, an animal model closer to human than rodents, and providing evidence that the expression of this taste-related signalling molecule is modified by feeding and fasting. G_αtran_-IR was predominantly in EECs, but the colocalization with CgA was not complete suggesting that G_αtran_-IR is also in non-EECs (likely brush cells), as it has been shown for G_αgust_ in the mouse 10. On the other hand, in the human colon 14 and pig small intestine 13, G_αgust_ has been reported exclusively in EECs. G_αtran_-IR cells had a different density throughout the gut, which was high in the stomach, decreased from the duodenum to the ileum, then increased from the caecum to the rectum. These findings are consistent with species and region differences and suggest that TRs exert distinct functions according to the gut region. Like G_αgust_, G_αtran_ mediates signals initiated by tastants acting at T1Rs and the T2Rs 7, 18, 19. Thus, G_αtran_ cells are likely to serve different chemosensitive modalities depending upon the luminal content and the TR stimulated 19. The colocalization of G_αtran_ with GHR in the stomach, and CCK and SOM in the small intestine is in agreement with previous studies in rodents and human 8, 9, 11, 14, and in EECs lines 20. GHR is an orexigenic peptide regulating energy balance homeostasis 21, GI motility and secretion 22, and feeding behaviour 23, in several species including pigs 24. CCK exerts a prominent role in satiety conveying signals elicited by nutrients (e.g. fats and proteins) *via* sensory nerve pathways to the brain 25. SOM inhibits gastric acid secretion, gastric emptying and smooth muscle contraction and GI hormone release 26. Thus, the colocalization of G_αtran_ with these peptides is consistent with an involvement of TRs in the control of satiety and food intake, energy balance metabolism and GI secretion and motility.

Food deprivation and refeeding alter the morphology of the weaned pig GI tract mucosa with fasting inducing mucosa atrophy in the upper small intestine and refeeding partially restoring it 27. We demonstrated that 24 h fasting and 24 h refeeding modified the number of G_αtran_-IR cells in most regions of the pig gut. The number of CgA-IR cells was not modified by fasting and refeeding in most regions with the exception of the caecum and descending colon, therefore it is unlikely that the reduction in G_αtran_-IR cells observed in fasted and in some regions also in refed animals is due to mucosa atrophy or lack of mucosal restoration following refeeding, although this possibility cannot be excluded. Fasting induces multiple changes in the EEC system such as increasing GHR and lowering GAS/CCK 28, 29 peptides that influence feeding behaviour and colocalize with G_αtran_-IR. Our results indicated that in the cardiac and pyloric mucosa, the number of G_αtran_/GHR cells is greater in normally fed compared with 24 h fasted piglets; similarly, the overall density of GHR-IR cells was lower in fasted than fed or refed animals. However, the increased G_αtran_/GHR-IR cell expression, as observed during refeeding state in our model, may not necessarily correspond to increased GHR plasma levels during fasting. A significant increase in plasma GHR was reported 30 in weaning pigs following 36 h fasting, with a decrease with 12 h fasting, indicating that the length of food deprivation affects GHR response. Animal ages might also affect hormonal responses to fasting, as young animals possess fewer energy reserves and less body fat, while having higher energy requirements in relation to rapid body growth 31. Our data showed a significant reduction in G_αtran_/CCK-IR cells and in CCK-IR cells overall in fasted and refed pigs compared with controls. This is in agreement with previous reports of a decrease in CCK plasma concentrations and mRNA expression during fasting, while returning to pre-fasting values after either 24 h refeeding in the rat small intestine 32 and 1 h refeeding in lactating sows 33. However, the reasons why in this study we did not detect an increase in G_αtran_/CCK-IR cells during refeeding remain to be elucidated. It is possible that factors such as caloric intake, type of diet and slaughter time after refeeding may contribute to explain why CCK cells do not return to pre-fasting values.

In summary, TRs and downstream molecules might exert a variety of functions ranging from sensing beneficial nutrients (e.g. sweet and umami), thus inducing secretion and motility to facilitate digestion, absorption and food intake, to detection of bitter, potentially harmful substances, thus inducing a defensive response. The latter could be in the form of inhibition of gastric emptying to reduce absorption, increase in intestinal secretion to facilitate elimination, vomiting or avoidance. Taste-related molecules in the distal colon and rectum could also serve as a line of defence against bacteria, which are particularly abundant in these regions. This is supported by the findings that quorum-sensing molecules produced by Gram-negative bacteria activate a GPCR-mediated signalling cascade in EEC lines, which is likely to involve T2R (Sternini C and Rozengurt E, unpublished). Further studies are required to better understand TR functions in the GI tract in response to feeding, including their regulation with specific dietary components in relationship to peptide release in different regions of the GI tract.

## Supporting information

**Data S1** Expression and regulation of a-transducin in the pig gastrointestinal tract
