# Supplementary material for: Expression and regulation of α-transducin in the pig gastrointestinal tract
Source: J Cell Mol Med. 2013 Feb 18;17(4):466–74. doi: 10.1111/jcmm.12026 (PMC3640731; doi:10.1111/jcmm.12026)

**Supplementary data**

**Expression and regulation of α-transducin in the pig gastrointestinal tract**

Mazzoni et al

**Western blot**

Pig stomach (pyloric mucosa), and small intestine (duodenum, jejunum) were collected, frozen in liquid nitrogen, and stored at −80°C. Tissues were later thawed and homogenized directly into a sodium dodecyl sulfate (SDS) lysis solution (Tris-HCl 62.5 mM, pH 6.8; SDS 2%, 5% glycerol) with 0.1 mM phenylmethylsulfonylfluoride. Protein content of cellular lysates was determined by a Protein Assay Kit (TP0300; Sigma-Aldrich, St. Louis, MO).

For G_αgust_ and G_αtrans_ antibodies specificity studies, aliquots containing 20 μg of proteins from tissue extracts were separated on NuPage 4-12% bis-Tris Gel (Gibco-Invitrogen, Paisley, UK) for 50 minutes at 200V. Proteins were then electrophoretically transferred onto a nitrocellulose membrane. Blots were washed in PBS and protein transfer was checked by staining the nitrocellulose membranes with 0.2% Ponceau Red. Following blocking treatment, the membranes were incubated at 4°C overnight with the respective antibodies in Tris-buffered saline-T20 (TBS-T20 20 mM Tris-HCl, pH 7.4, 500 mM NaCl, 0.1% T-20): anti-G_αgust_ rabbit polyclonal antibody at 1:300; anti- G_αtrans_ rabbit polyclonal antibody at 1:500.

For GAS/CCK antibody specificity studies, aliquots containing 30 μg of proteins from tissue extracts were separated on Novex 18% Tris-Glycine Gel (Gibco-Invitrogen, Paisley, UK) for 90 minutes at 125V. Proteins were then electrophoretically transferred onto a nitrocellulose membrane. Blots were washed in PBS and protein transfer was checked by staining the nitrocellulose membranes with 0.2% Ponceau Red. Following blocking treatment, the membranes were incubated at 4°C overnight with anti-GAS/CCK mouse monoclonal antibody at1:1,000.

Following several washings with PBS-T20, the membranes were incubated with the secondary biotin-conjugated antibody and then with a 1:1,000 dilution of an anti-biotin horseradish peroxidase linked antibody. Western Blots were developed using chemiluminescent substrate (Super Signal West Pico Chemiluminescent Substrate, Pierce Biotechnology, Rockford, IL) according to the manufacturer's instructions. The intensity of luminescent signal of the bands was acquired by Fluor-STM Multimager using the Quantity One Software (Bio-Rad Laboratories, Hercules, CA).

For G_αtrans_ antibody, a major band of ≈45 kDa (theoretical molecular weight 40 kDa in human) was present in extracts from the stomach and intestine (Fig. 1). As positive control tissue we used mouse brain.

For G_αgust_ antibody, a major band of ≈40 kDa (theoretical molecular weight 40 kDa in human) was present in extracts from the stomach and intestine (Fig. 2). As positive control tissue we used mouse brain.

Different molecular forms of CCK have been described deriving from enzymatic cleavage of a precursor peptide of 115 AA (UNIPROT P06307) so the expected molecular weight of CCK is between 4 and 20 kDa. Fig. 3 shows a major band showing with a molecular weight of ≈15 Da in the intestine. The smallest form of CCK (10 different chains from 58 to 5 AA) could not be identified probably due to the very low amount of each component present in the tissue.

Fig. 1 Western Blot of G_αtrans_ antibody shows a major band close to the theoretical molecular weight (~45 kDa). Right lane: Molecular Weight Marker; the numbers inside this lane indicate the molecular weight. The images were slightly adjusted in brightness and contrast to match background. Lane 1 Mouse brain (positive control); lane 2 pig stomach; lane 3 pig intestine).


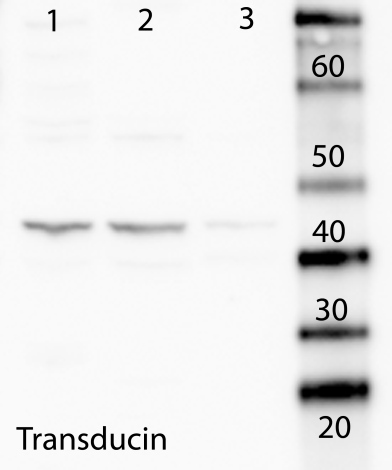


Fig. 2 Western Blot of G_αgust_ antibody shows a major band close to the theoretical molecular weight (~40 KDa). Right lane: Molecular Weight Marker; the numbers inside this lane indicate the molecular weight. The images were slightly adjusted in brightness and contrast to match background. Lane 1, mouse brain (positive control); lane 2 pig intestine; lane 3 pig stomach.


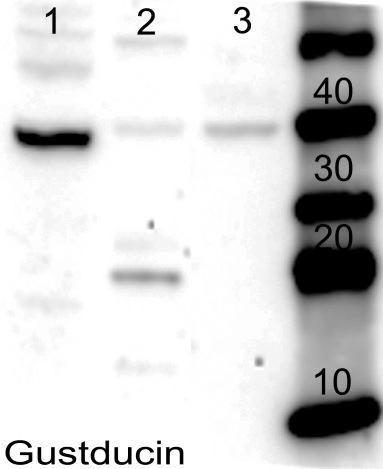


Fig. 3 Western Blot of GAS/CCK antibody shows a major band close to the theoretical molecular weight (~15) in the pig intestine. Left lane: Molecular Weight Marker; the numbers inside this lane indicate the molecular weight. The images were slightly adjusted in brightness and contrast to match background.


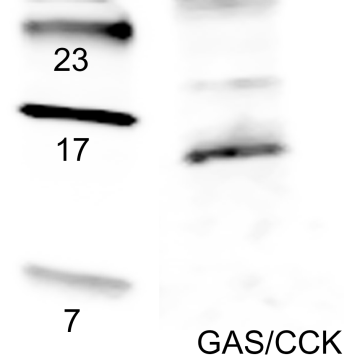

Supplement: Supplementary file 1 [file jcmm0017-0466-SD1.docx]
